# Effect of MR Imaging Contrast Thresholds on Prediction of Neoadjuvant Chemotherapy Response in Breast Cancer Subtypes: A Subgroup Analysis of the ACRIN 6657/I-SPY 1 TRIAL

**DOI:** 10.18383/j.tom.2016.00247

**Published:** 2016-12

**Authors:** Wen Li, Vignesh Arasu, David C. Newitt, Ella F. Jones, Lisa Wilmes, Jessica Gibbs, John Kornak, Bonnie N. Joe, Laura J. Esserman, Nola M. Hylton, ACRIN 6657 Trial Team and I-SPY Investigators Network

**Affiliations:** 1 Departments of Radiology and Biomedical Imaging,; 2 Epidemiology and Biostatistics, and; 3 Surgery, University of California San Francisco, San Francisco, California

**Keywords:** MRI, breast cancer, neoadjuvant, contrast enhancement

## Abstract

Functional tumor volume (FTV) measurements by dynamic contrast-enhanced magnetic resonance imaging can predict treatment outcomes for women receiving neoadjuvant chemotherapy for breast cancer. Here, we explore whether the contrast thresholds used to define FTV could be adjusted by breast cancer subtype to improve predictive performance. Absolute FTV and percent change in FTV (ΔFTV) at sequential time-points during treatment were calculated and investigated as predictors of pathologic complete response at surgery. Early percent enhancement threshold (PEt) and signal enhancement ratio threshold (SER_t_) were varied. The predictive performance of resulting FTV predictors was evaluated using the area under the receiver operating characteristic curve. A total number of 116 patients were studied both as a full cohort and in the following groups defined by hormone receptor (HR) and HER2 receptor subtype: 45 HR+/HER2−, 39 HER2+, and 30 triple negatives. High AUCs were found at different ranges of PE_t_ and SER_t_ levels in different subtypes. Findings from this study suggest that the predictive performance to treatment response by MRI varies by contrast thresholds, and that pathologic complete response prediction may be improved through subtype-specific contrast enhancement thresholds. A validation study is underway with a larger patient population.

## Introduction

Breast cancer, the most common type of cancer among women, is a heterogeneous disease comprising subtypes with different biology, prognosis, and treatment outcome. Breast cancer can be classified into subtypes based on the hormone receptor (HR) status, including both estrogen and progesterone receptors, and human epidermal growth factor receptor 2 (HER2) expression to inform treatment decisions (1, 2). These breast cancer subtype classifications also have implications for disease-free survival and relapse (3). Further understanding of subtype-specific response and effective monitoring by imaging may provide means for early therapeutic intervention, leading to better outcomes (4).

Magnetic resonance imaging (MRI) is one of the most accurate imaging tools used to monitor and predict treatment response for patients undergoing chemotherapy (5–14). However, the predictive performance varies between different quantitative measurements derived from MRI, and by variations in the parameters that define those measurements. Previous studies have found that the tumor volume measured using MRI for patients undergoing preoperative chemotherapy has strong association with recurrence-free survival (13, 15, 16), and the association is influenced by the threshold settings of 2 contrast enhancement parameters (17). Another recent study has demonstrated that the influence varied in HR/HER2− defined breast cancer subtypes (18).

A standardized MRI-derived volume calculation procedure was used in the I-SPY 1 TRIAL (*I*nvestigation of *S*erial Studies to *P*redict *Y*our *T*herapeutic *R*esponse with *I*maging *A*nd mo*L*ecular *A*nalysis) imaging sub-study: *A*merican *C*ollege of *R*adiology *I*maging *N*etwork (ACRIN) 6657. This procedure used empirically determined, site-specific analysis parameters, specifically an early time-point percent enhancement threshold (PE_t_) and a signal enhancement ratio threshold (SER_t_) for calculation of a functional tumor volume (FTV) for patients undergoing neoadjuvant (preoperative) chemotherapy (NACT) for breast cancer. FTV was shown to be predictive of both treatment response, as measured by pathological complete response (pCR) (15), and of recurrence-free survival (16) in the study population.

In the current study, we explored how the pCR prediction performance of FTV varies over a wide range of PE_t_ and SER_t_, for different serial time-point MRI scans during the NACT course, and for different patient cohorts determined by HR and HER2 status. We show that the predictive performance to treatment response by MRI varies by contrast thresholds, and that the pCR prediction may be improved through subtype-specific contrast enhancement thresholds.

## Methodology

### Patient Population

**Figure 1.**
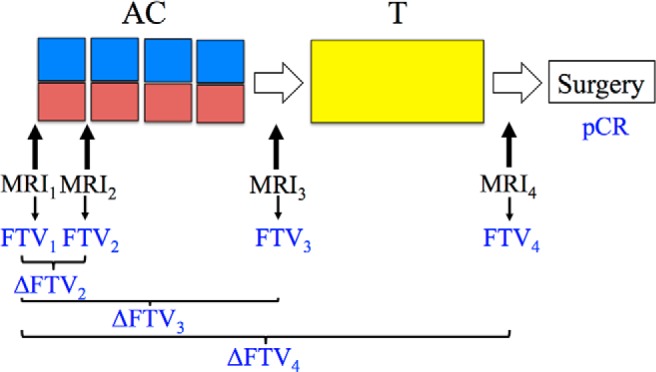
I-SPY 1 TRIAL and ACRIN 6657 study schema. Patients received anthracycline–cyclophosphamide (AC)- and taxane (T)-based chemotherapy and had pathological complete response (pCR) assessed at surgery. Magnetic resonance imaging (MRI) was performed at 4 treatment time-points, and the corresponding functional tumor volume (FTV) was generated. Percent change of FTV (ΔFTV) compared with FTV_1_ was also calculated at each treatment time-point after baseline.

In total, 237 women with breast tumors sized ≥3 cm evaluated by either clinical examination or imaging were enrolled between 2002 and 2006 at 9 institutions in the USA. All patients provided written consent. As shown in Figure 1, 4 MRI examinations were conducted for each patient at the following time-points: before starting anthracycline–cyclophosphamide (AC) chemotherapy (MRI_1_); at least 2 weeks after the first cycle and before the second AC cycle (MRI_2_); between regimens if taxane was given (MRI_3_); and following the completion of chemotherapy but before surgery (MRI_4_). A subset of 116 patients that had image data from all 4 MRIs, pathological outcomes, and HR/HER2 status were analyzed for this retrospective study. The detailed design and previous findings of I-SPY 1 TRIAL/ACRIN 6657 have been previously published (15, 16, 19, 20).

### Determination of Breast Cancer Subtype

HR status and HER2 receptor expression were determined by pretreatment core biopsy, using immunohistochemistry (IHC) and Allred score at study sites. The HER2 status was determined by IHC and/or fluorescence in situ hybridization assays. Unlike HRs, HER2 testing (IHC and fluorescence-in situ hybridization assays) was performed locally at study sites and centrally at the University of North Carolina (19). Estrogen or progesterone receptor was positive if Allred score was ≥3, that is, ≥3% cells stained positive. HER2 was positive if it was tested positive at either a local or a central laboratory. The following 3 subtype groups were defined: HR+HER2−; HER2+ (HR either positive or negative); and triple-negative breast cancer (TNBC, ie, HR−HER2−) tumors.

### Evaluation of Pathological Response

The pCR was considered as the surrogate end point of NACT and was defined as the absence of residual invasive disease in the breast and axillary lymph nodes at surgery (19). By this definition, patients were classified into 2 groups at the end of NACT as follows: pCR and non-pCR (residual invasive cancer). In I-SPY 1/ACRIN 6657, pCR was evaluated locally by each institution's pathologist immediately after surgery. In the event of a patient declining surgery, there was no pCR status for that patient.

### Image Acquisition

Each patient had 4 MRIs (Figure 1) at their participating site using a 1.5 T scanner and dedicated 4- or 8-channel breast radiofrequency coil. Imaging was performed with the patient in the prone position with an intravenous catheter inserted in the antecubital vein or hand. The image acquisition protocol was prespecified, and it included a localization scan and T2-weighted sequences, followed by a contrast-enhanced T1-weighted series. For the contrast-enhanced T1-weighted series, high spatial resolution (in-plane spatial resolution, ≤1 mm), 3-dimensional fat-suppressed T1-weighted imaging of the symptomatic breast was performed using a gradient-echo sequence with the following parameters: repetition time = 4.5 milliseconds, flip angle ≤45°, field of view = 16–18 cm, minimum matrix = 256 × 192, sections = 64, and section thickness ≤2.5 mm.

All imaging tests were performed unilaterally over the symptomatic breast and in the sagittal orientation. Imaging time for the T1-weighted sequence was between 4.5 and 5 minutes, with one data set acquired before injection of a gadolinium-based contrast agent and repeated 2–4 times immediately after injection. Interimaging delays were added as needed to result in postcontrast administration temporal sampling between 2 minutes 15 seconds and 2 minutes 30 seconds for early-phase images and between 7 minutes 15 seconds and 7 minutes 45 seconds for delayed-phase images.

### Functional Tumor Volume Measurement

**Figure 2.**
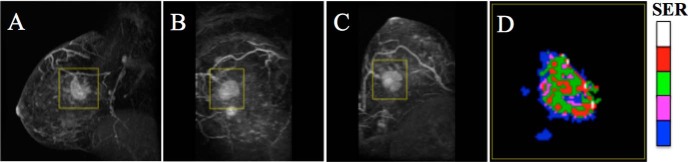
The maximum intensity projection (MIP) dynamic contrast-enhanced (DCE)-MRI images showing the symptomatic breast. Images are shown in orthogonal views: sagittal (A), coronal (B), and axial (C). Yellow rectangular boxes were placed manually in 2 MIPs to enclose the tumor. The SER map from a representative section from the sagittal view after applying the default PE threshold at 70% (D). All voxels with SER ≥ 0 were color coded as follows: blue, 0 ≤ SER < 0.9; purple, 0.9 ≤ SER < 1.0; green, 1.0 ≤ SER < 1.3; red, 1.3 ≤ SER ≤ 1.75; and white, SER > 1.75).

Following each MRI examination, image data were transferred to the ACRIN Core Lab for central archival and subsequently to the University of California at San Francisco for image analysis. All images were analyzed using in-house software developed in the IDL programming environment (ITT Visual Information Solutions, Boulder, Colorado) (21). For each dynamic contrast-enhanced (DCE-) MRI acquisition, a region of interest (ROI) encompassing the primary tumor as determined by signal enhancement was manually defined by a trained research associate by placing rectangular boxes on orthogonal maximum intensity projection images created from the early postcontrast scan (Figure 2A–C). Background air regions and suppressed fat regions were masked out using an automatically determined intensity threshold applied to the precontrast image.

The FTV was then measured using the signal enhancement ratio method within the ROI (22). The volumes of image voxels within the ROI that met PE_t_ and SER_t_ were summed to compute FTV, constrained by a minimum number of connected voxels to eliminate isolated voxels. PE and SER were calculated at each voxel as follows: PE = 100% × (S_1_ − S_0_)/S_0_ and SER = (S_1_ − S_0_)/(S_2_ − S_0_), where S_0_, S_1_, and S_2_ were signal intensities at precontrast, early contrast, and late postcontrast, respectively, collected during the DCE-MRI scan (23). A cutoff PE_t_ was first applied followed by a connectivity test to create an enhanced tissue mask. SER was then calculated for all voxels in the mask (Figure 2D), and SER_t_ was applied to determine which voxels to include in the FTV. In ACRIN 6657, PE_t_ was nominally set at 70% and adjusted empirically for each site to qualitatively reflect the extent of tumor and to account for unexpected variability in MRI systems and imaging parameters. SER_t_ was set to be zero across all participant sites in the primary aim analysis of the trial. All magnetic resonance images from a given site were processed using the same site-specific PE_t_. To study the effect of PE_t_/SER_t_ setting, we recalculated FTV by varying these 2 thresholds. PE_t_ was changed from 30% to 200% in steps of 10% and SER_t_ from 0 to 2 in steps of 0.2. FTV was recalculated at each MR examination as follows: baseline (FTV_1_), early treatment (FTV_2_), inter-regimen (FTV_3_), and before surgery (FTV_4_). Percent change of FTV was defined as the change in FTV relative to the baseline FTV_1_ value (ΔFTV_n_ = 100% × (FTV_n_ − FTV_1_)/FTV_1_, n = 2, 3, 4).

### Statistical Analysis

FTV measurements were calculated for each pair of PE_t_/SER_t_ values, and associations with pCR were evaluated using receiver operating characteristic (ROC) curve analysis. The area under the ROC curve (AUC) was estimated to provide a measure of predictor quality. In the statistical model, patients with pCR were considered as controls (negative outcome) and those with non-pCR were considered as cases (positive outcome). For each PE_t_/SER_t_ pair, the AUC was estimated in the full cohort and separately in each specific breast cancer subtype. The AUCs were then mapped as a surface plot on the axes of PE_t_ (range, 30%–200%) and SER_t_ (range, 0–2) for each FTV measurement. Higher AUC indicates “stronger association” between the measurement and pCR status. The optimized PE_t_/SER_t_ was selected as having the maximum AUC over the map of PE_t_/SER_t_ combinations. The processes of calculating FTV for each specific PE_t_/SER_t_ pair, estimating AUCs, and selecting optimized PE_t_/SER_t_ based on AUC values were performed automatically after the ROI was defined.

Because of the small sample size, it was not feasible to perform cross validation and hence AUCs and predictive accuracy estimates will be subject to overfitting. An optimal cutoff point was chosen as closest to sensitivity = 100% and specificity = 100% on the ROC curve (24). Data processing and optimization were performed in Matlab (R2012b 64bit for Mac, MathWorks Inc., Natick, Massachusetts), and all statistical analyses were conducted using the R statistical analysis software package and the pROC library (25, 26). Data are expressed as median with interquartile range. All tests were performed at the *P* < .05 level, and all results are provided with estimates, 95% confidence intervals (CIs), and *P* values if appropriate.

## Results

A cohort of 116 patients was analyzed. The status of HR and HER2 was available for primary tumors in 115 patients (99%). Characteristics of patients with and without pCR are described in Table 1.

**Table 1.** Patient Characteristics of the Full Cohort (n = 116)

Characteristics	pCR (n = 34)	Non-pCR (n = 82)	*P*^a^
Age, Median (range)	47 (31−69)	49 (28−67)	0.3
Tumor size (cm), median (IQR)	5.0 (4.1−6.0)	5.5 (4.0−7.1)	0.25
Menopausal status			0.86
Premenopausal	16 (13.8)	35 (30.2)	
Postmenopausal	12 (10.3)	33 (28.4)	
Undetermined	6 (5.2)	14 (12.1)	
Surgery method			0.69
Mastectomy	16 (13.8)	43 (37.1)	
Breast-conserving surgery	18 (15.5)	40 (34.5)	
Histological subtype			1
Invasive ductal carcinoma	30 (25.9)	66 (56.9)	
Invasive lobular carcinoma	2 (1.7)	5 (4.3)	
Mixed ductal and lobular carcinoma	0 (0.0)	1 (0.9)	
Other	2 (1.7)	5 (4.3)	
HR status			**0.004**
Negative	22 (19.0)	28 (24.1)	
Positive	12 (10.3)	54 (46.6)	
HER2 status			0.096
Negative	18 (15.5)	58 (50.0)	
Positive	16 (13.8)	23 (19.8)	
Missing	0 (0.0)	1 (0.9)	
Axillary lymph node status at initial staging			**0.01**
Negative	24 (20.7)	27 (23.3)	
Positive	19 (16.4)	55 (47.4)	
NA	1 (0.9)	0 (0.0)	
HR/HER2 status			
HR−/HER2− (TNBC)	11 (9.5)	19 (16.4)	**0.01**
HR+/HER2−	6 (5.2)	39 (33.6)	
HER2+	16 (13.8)	23 (19.8)	
Missing	1 (0.9)	0 (0.0)	

Data in parentheses are percentages.

^a^ Wilcoxon *P* value was used for continuous variables; and Fisher exact test was used for categorical variables. Numbers in bold are significant (*P* < 0.05).

### Effect of Varying PE_t_/SER_t_ on Predicting pCR

Analyses of surgical samples revealed pCR in 34 patients (29%). The remaining 82 patients (71%) did not achieve pCR (non-pCR). Among 45 patients with HR+/HER2− breast cancer, only 6 (estimated percentage, 13%, with 95% CI of 5% to 27%) achieved pCR. Sixteen HER2+ patients out of 39 (estimated percentage: 41%, with 95% CI of 26% to 58%) achieved pCR and 11 out of 30 patients (estimated percentage: 37%, with 95% CI of 20% to 56%) achieved pCR in the TNBC subgroup.

**Figure 3.**
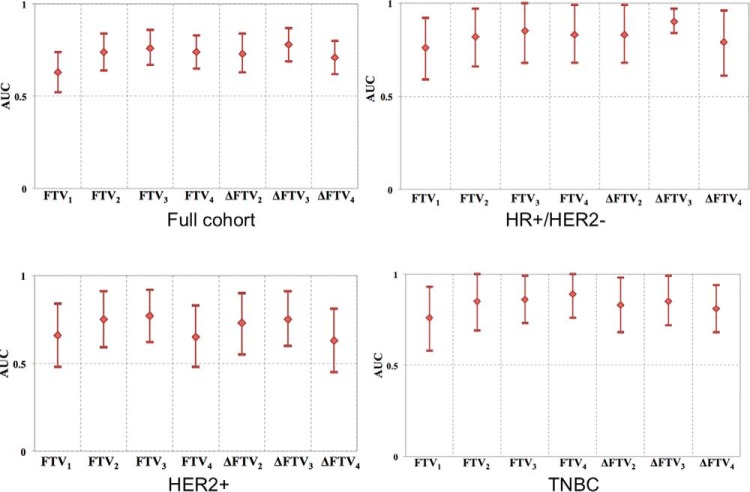
Maximum area under the receiver operating characteristic curve (AUCs) observed for FTV prediction of pCR. Plots were generated for patients in the full cohort and in HR+/HER2−, HER2+, and TNBC cohorts, separately. Each AUC was plotted with 95% confidence interval (CI).

Figure 3 shows the highest AUCs observed for FTV measurements at different treatment time-points for the full cohort and by breast cancer subtype. In general, AUCs evaluated in subtypes were estimated to be higher than those in the full cohort, of which triple negatives had the highest estimated AUCs. In addition, absolute FTVs and ΔFTVs at MRI_2_ and MRI_3_ showed higher AUCs than those measured at MRI_1_ and MRI_4_. The estimated AUC at ΔFTV_3_ in the HR+/HER2− subgroup was among the highest with a narrow confidence interval. Although ΔFTV_3_ showed no significance difference relative to other FTV predictors in the full cohort and other subtypes, we focused our contrast threshold comparison between subgroups using ΔFTV_3_ as a predictor.

**Figure 4.**
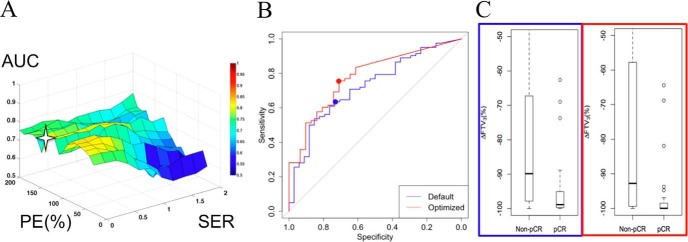
Effects of percent enhancement threshold (PE_t_) and signal enhancement ratio threshold (SER_t_) on the subsequent ΔFTV_3_ association with pCR and non-pCR in the full cohort of 116 patients. The 3-dimensional (3D) surface map of estimated AUCs tested with all PE_t_ and SER_t_ combinations (A). The star indicates where maximum AUC is observed: PE_t_ = 130% and SER_t_ = 0.0. The receiver operating characteristic (ROC) curves when ΔFTV_3_ was calculated using 130%/0 (in red) versus the default (in blue) (B). The estimated AUC for the blue curve is 0.73 (95% CI, 0.63–0.82) and that for the red curve is 0.78 (95% CI, 0.69–0.87). Dots on ROCs indicate the optimal cutoff point for diagnostic tests. Box plots of ΔFTV_3_ values in patients with pCR and those without pCR (non-pCR) calculated with default (on the left) versus 130%/0.0 (on the right) (C).

**Figure 5.**
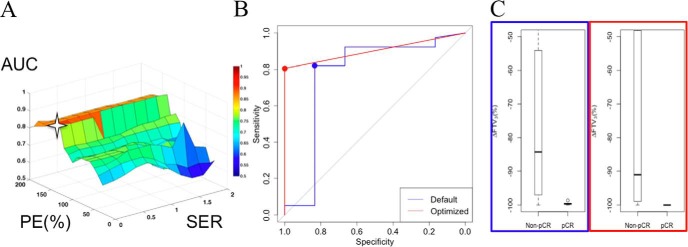
Effects of PE_t_ and SER_t_ on the subsequent ΔFTV_3_ association with pCR and non-pCR in the HR+/HER2− subtype of 45 patients. The 3D surface map, with the star indicating where maximum AUC is observed: PE_t_ = 130% and SER_t_ = 0 (A). The ROC curves of using 130%/0 (in red) versus the default (in blue) (B). Estimated AUC for the blue curve is 0.77 (95% CI, 0.48–1.00) and that for the red curve is 0.90 (95% CI, 0.84–0.97) (B). Box plots of ΔFTV_3_ values in patients with pCR and those without pCR (non-pCR) calculated with default (on the left) versus 130%/0 (on the right) (C).

**Figure 6.**
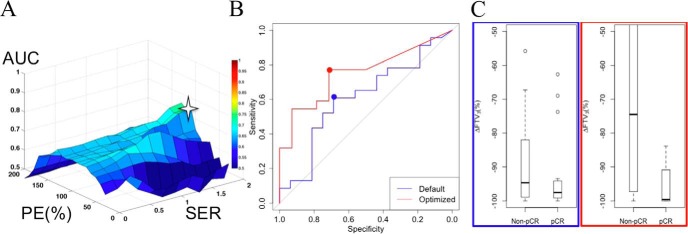
Effects of PE_t_ and SER_t_ on the subsequent ΔFTV_3_ association with pCR and non-pCR in the HER2+ subtype of 39 patients. The 3D surface map, with the star indicating where maximum AUC is observed: PE_t_ = 130% and SER_t_ = 2.0 (A). The ROC curves of using 130%/2.0 (in red) versus the default (in blue) (B). Estimated AUC for the blue curve is 0.61 (95% CI, 0.42–0.80) and that for the red curve is 0.75 (95% CI, 0.60–0.91) (B). Box plots of ΔFTV_3_ values in patients with pCR and those without pCR (non-pCR) calculated with default (on the left) versus 130%/2.0 (on the right) (C).

**Figure 7.**
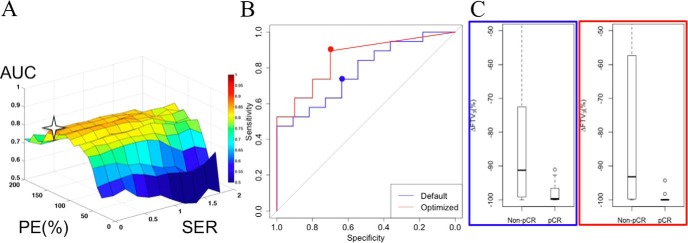
Effects of PE_t_ and SER_t_ on the subsequent ΔFTV_3_ association with pCR and non-pCR in 30 patients with TNBC. The 3D surface map, with the star indicating where maximum AUC is observed: PE_t_ = 140% and SER_t_ = 0.0 (A). The ROC curves of using 140%/0.0 (in red) versus the default (in blue) (B). Estimated AUC for the blue curve is 0.78 (95% CI, 0.61–0.95) and that for the red curve is 0.85 (95% CI, 0.72–0.997). Box plots of ΔFTV_3_ values in patients with pCR and those without (non-pCR) calculated with default (on the left) versus 140%/0.0 (on the right) (C).

In the full cohort among all PE_t_/SER_t_ combinations, ΔFTV_3_ exhibited higher estimated AUCs (≥0.75) at 70% ≤ PE_t_ ≤ 140% and lower range of SER_t_ (0.0−1.0) (Figure 4A). Within specific subtypes, differential effect of varying PE_t_/SER_t_ on the prediction of using ΔFTV_3_ for pCR was observed. In the HR+/HER2− subgroup (Figure 5A), higher estimated AUCs occurred at higher PE_t_ ranging from 120% to 200% across the entire range of SER_t_ (0.0−2.0). In the HER2+ subgroup (Figure 6A), high AUCs occurred at PE_t_ from 70% to 140% and at SER_t_ from 1.0 to 2.0. In the TNBC subtype (Figure 7A), higher estimated AUCs also occurred at a PE_t_ range of 60% to 150% and across the entire range of SER_t_ (0.0−2.0).

To demonstrate the improved discrimination of pCR versus non-pCR using optimized PE/SER thresholds, we examined ΔFTV_3_ in the full cohort and in breast cancer subtypes. Table 2 shows diagnostic performance for cutoff points selected from ROC curves **(**Figures 4–7B). In the full cohort, inconsistent effects on sensitivity and specificity were observed, whereas a consistent improvement was shown in subtypes. Table 3 shows ΔFTV_3_ values and differences between patients with pCR and those without pCR (non-pCR) (Figures 4–7C). *P* values in Table 3 were estimated by likelihood ratio test. Lower *P* values at optimized PE_t_/SER_t_ in subtypes may indicate that ΔFTV_3_ calculated by optimized PE_t_/SER_t_ has stronger predictive value for pCR than the default. Odds ratios were also estimated to be larger using optimized than default thresholds.

**Table 2.** Diagnostic Performance for Optimal Cutoff Point of ΔFTV_3_

Patient Population	PE_t_/SER_t_	Cutoff Point	Sensitivity (95% CI)	Specificity (95% CI)
Full cohort	Default	−95%	63% (52, 74)	74% (56, 87)
	130%/0	−99.4%	76% (65, 84)	71% (52, 86)
HR+/HER2−	Default	−97.8%	79% (64, 91)	83% (36, 99.5)
	130%/0	−99.3%	78% (61, 90)	100% (48, 100)
HER2+	Default	−96.5%	61% (39, 80)	69% (41, 89)
	130%/2	−97.3%	77% (55, 92)	71% (42, 92)
Triple negative	Default	−98.9%	74% (49, 91)	64% (31, 89)
	140%/0	−99.98%	89% (67, 99)	70% (35, 93)

**Table 3.** Median ΔFTV_3_ Values in Patients With and Without pCR in Optimized Versus Default PE_t_/SER_t_ Setting

Patient Population	PE_t_/SER_t_	ΔFTV_3_ (IQR)		Difference (95% CI)	*P* Value
		pCR	Non-pCR		
Full cohort	Default	−99% (−99.9, −73.5)	−88.8% (−97.8, −62.5)	−6% (−2, −15)	<0.001
	130%/0	−100% (−100, −98.5)	−92.8% (−99.4, −58.6)	−5% (−1, −14)	<0.001
HR+/HER2−	Default	−99.6% (−99.8, −98.9)	−83.4% (−97, −53.8)	−12% (−0.1, −45)	0.3
	130%/0	−100% (−100, −98.5)	−92.8% (−99.4, −58.6)	−9% (−0.7, −54)	<0.001
HER2+	Default	−97.5% (−99.1, −94.4)	−94.6% (−98.9, −82)	−2% (1, −11)	0.4
	130%/2	−99.2% (−100, −88.8)	−74.5% (−96.6, −23.7)	−16% (−8, −59)	0.004
Triple negative	Default	−99.7% (−99.9, −96.6)	−91.3% (−99.1, −72.5)	−6% (−0.2, −25)	0.002
	140%/0	−100% (−100, −99.9)	−93.2% (−99.8, −57.4)	−7% (−0.1, −48)	0.01

**Figure 8.**
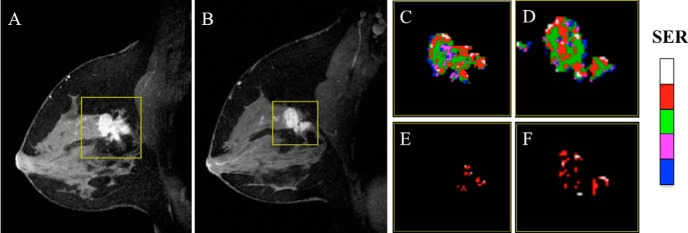
A representative clinical case of varying PE_t_/SER_t_ in FTV measurement in a patient with HR+/HER2− subtype. A sagittal section at MRI_1_ (A). A sagittal section at MRI_2_, at roughly the same location as (A) (B). Yellow boxes are regions of interest (ROIs) encompassing the whole tumor. Enlarged tumor ROI in (A) and (B) when PE_t_/SER_t_ are set at default values (70%/0.0) (C) and (D). FTV_1_ = 5.31 cc and FTV_2_ = 5.13 cc. Same tumor sections as shown in (C) and (D) but with PE_t_/SER_t_ optimized for HR+/HER2− subtype specifically: 140%/1.4 (E) and (F). The subsequent FTV_1_ = 1.33 cc and FTV_2_ = 2.58 cc. Tumor voxels in (C)–(F) are shown with SER ≥ 0 that are color coded as follows: blue, 0 ≤ SER < 0.9; purple, 0.9 ≤ SER < 1.0; green, 1.0 ≤ SER < 1.3; red, 1.3 ≤ SER ≤ 1.75; white, SER > 1.75).

Figure 8 shows an example of the effect of PE_t_/SER_t_ on tumor voxels and subsequent FTV calculations in DCE-MRI. In this example, a 38-year-old female patient with a tumor sized 4 cm was enrolled in the I-SPY 1 TRIAL. The tumor was identified to be HR+/HER2− before treatment. The patient received AC- and taxane-based chemotherapy, and she did not achieve pCR at the completion of the treatment.

The effect of varied PE_t_/SER_t_ on estimated AUCs for FTV_2_, ΔFTV_2_, and FTV_3_ is shown in the supplement of this paper. When comparing absolute measures FTV_2_ and FTV_3_ with percent change ΔFTV_2_ and ΔFTV_3_ (Figure 4–7A), the absolute measurements are more reliable in predicting pCR over a wider range of PE_t_/SER_t_. In HR+/HER2− subtype, higher estimated AUCs were observed at high PE_t_ in all FTV measurements. Estimated AUCs for HER2+ are generally lower than HR+/HER2− and TNBC, which can also be observed in Figure 3. A mixed effect of PE_t_/SER_t_ in TNBC was observed when high AUCs were found at a higher range of PE_t_ for FTV_2_, FTV_3_, and ΔFTV_3_ but at lower range of PE_t_ for ΔFTV_2_.

## Discussion

In this study, the impact of PE and SER thresholds on FTV prediction of neoadjuvant treatment response was retrospectively investigated using data from the I-SPY 1 TRIAL/ACRIN 6657. In that study, default PE_t_ and SER_t_ levels were used in the FTV calculations that were empirically set by visual evaluation of DCE images. In this paper, we present a semiautomated method to customize the PE_t_ and SER_t_ parameters, particularly for breast cancer subtypes, to account for the heterogeneity of tumor biology as reflected in imaging biomarkers. Through the optimization framework of this study, we seek to better understand the enhancement patterns of individual breast cancer subtypes and the association between enhancement measurements and pathologic outcomes of NACT.

Various forms of FTV have been investigated and compared previously to test predictive performance measured at different time-points during the treatment. Previous work on the ACRIN 6657 study reported AUCs of FTV ratios at MRI_2_, MRI_3_, and MRI_4_ relative to MRI_1_ in predicting pCR using the default PE_t_/SER_t_ (15). In a study using earlier data from a pilot cohort of 64 patients imaged at a single center (18), the effect of varying PE_t_/SER_t_ on FTV and ΔFTV was investigated. The percent change in FTV over the entire course of treatment from baseline to before surgery (ΔFTV_f_) was the predictor with the highest hazard ratio in the full cohort and the HR+/HER2− and HER2+ subgroups, whereas the absolute presurgical FTV (FTV_f_) was the highest for the TNBC subtype. In this study, FTV was calculated at MRI_1–4_ and percent change of FTV at MRI_2, 3, 4_. Although the inter-regimen metrics FTV_3_/ΔFTV_3_ generally showed the higher estimated AUCs, AUCs of the presurgery values FTV_4_/ΔFTV_4_ varied across patient cohorts (Figure 3). Meanwhile, FTV_2_/ΔFTV_2_ had similarly high AUCs as FTV_3_/ΔFTV_3_ across all patient cohorts except HR+/HER2−. Given the small sample size, these observations are limited to this study only. Cross validation is needed to confirm it in a general population.

PE and SER measure the signal enhancement characteristics of pre- and postcontrast injection during DCE-MRI (22). These 2 basic measurements and their thresholds may have a profound effect on the subsequent FTV calculation and, hence, its predictive performance of response in breast cancer subtypes during the treatment course. The current study showed that higher AUCs were observed at higher PE_t_ when absolute FTV was used to predict pCR in HR+/HER2− subtype. A similar finding was observed in the HR+ subgroup in a previous study (18), indicating that higher PE_t_ may better discriminate regions of malignant tumor from the high background parenchymal enhancement often found in HR+ patients (27–30). High SER value is indicative of tissue with a strong contrast washout characteristic and is generally associated with malignancy (31). Many studies have reported that TNBC shows a malignant enhancement pattern on DCE-MRI (32–36). Li et al. reported that postchemotherapy tumor volume with high SER had a statistically significant association with disease recurrence (37). Among breast cancer subtypes in this study, HER2+ was most affected by SER_t_ at FTV_3_ and ΔFTV_3_. Higher AUCs were observed at higher SER_t_, suggesting distinct biology and microenvironment within the HER2+ tumor that differ from other subtypes.

Compared with HR+/HER2− and TNBC, HER2+ had lower AUCs. This may be because of the heterogeneity within this subgroup, which included both HR+ or HR−. Because of the small sample size, we could not further subset this group into HR+/HER2+ and HR−/HER2+. The heterogeneity within this subtype may limit the effectiveness of changing PE_t_/SER_t_ to improve AUC. Furthermore, although trastuzumab is the current standard treatment for HER2+ patients, it was not used routinely in the timeframe of this study. Only 13 of 39 HER2+ patients received trastuzumab therapy. This adds complexity to this subtype and may have also created bias in our results. Because of the small sample size, we did not exclude these patients.

The presented retrospective study has a few limitations. First, the image quality may not be consistent in our patient cohort. Imaging data in this study were collected from a multicenter clinical trial and were acquired from 7 participating sites in the USA. The default PE_t_/SER_t_ setting varied across sites, and we only studied the subsequent calculated FTVs by applying subtype-specific thresholds. Second, the sample size is too small to perform any kind of validation (or cross validation) of the optimization model. The highest AUCs found in the full cohort and in subtypes may therefore overestimate the true optimal values. Further study on an independent cohort should therefore be performed to evaluate the extent to which our estimated AUCs represent generalizable improvement in predictive values. Again because of the relatively smaller sample sizes, AUCs estimated in subtypes have wider CIs compared with those estimated in the full cohort. In this study of 116 patients, we were unable to evaluate other factors such as age, tumor size, and axillary lymph node status. Third, the treatment was not the same for all subtypes. The data set was acquired between May 2002 and March 2006. All patients in our cohort had AC and taxane therapy before surgery, and one-third of HER2+ patients received additional trastuzumab. These different treatments can affect the predictive performance of ΔFTV with or without optimization. Finally, HER2+ subtype comprised both HR+/HER2+ and HR−/HER2+, posing potential heterogeneity in the analysis. In our planned future study with a larger cohort, the HR+/HER2+ and HR−HER2+ subsets will be separately analyzed.

### Supplemental Materials

Supplemental Figure 1:

Supplemental Figure 2:

Supplemental Figure 3:
