# Supplementary material for: Effect of MR Imaging Contrast Thresholds on Prediction of Neoadjuvant Chemotherapy Response in Breast Cancer Subtypes: A Subgroup Analysis of the ACRIN 6657/I-SPY 1 TRIAL
Source: Tomography. 2016 Dec;2(4):378–87. doi: 10.18383/j.tom.2016.00247 (PMC5214452; doi:10.18383/j.tom.2016.00247)
Supplement: Supplemental Figure 1: [file tom-00247-16-s001.pdf]

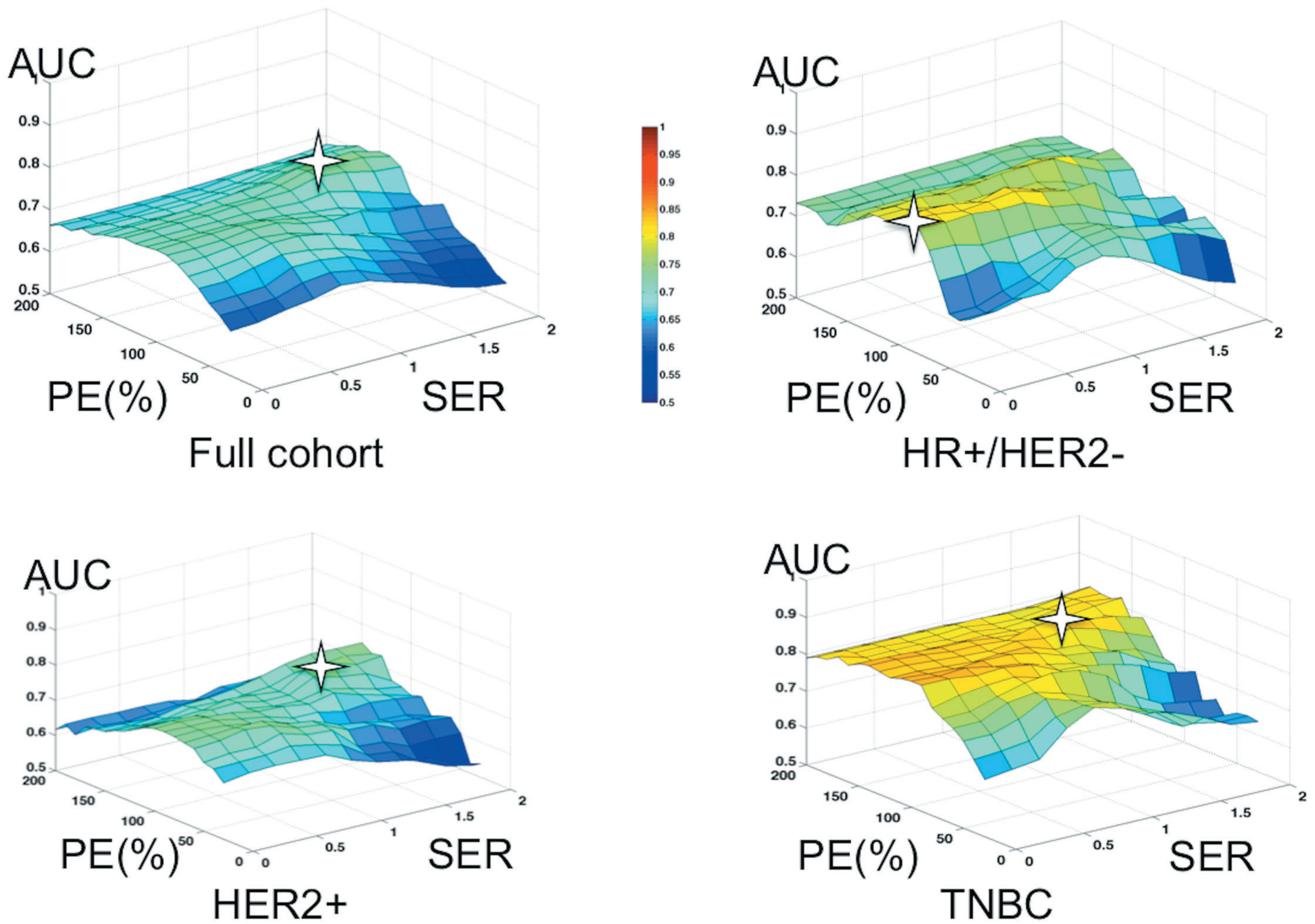

Figure S1. Surface plots of estimated AUCs for FTV_2 on PE_t/SER_t map. AUCs are estimated for FTV_2 calculated with all PE_t/SER_t combinations tested. The surfaces are plotted for estimations in the full cohort and separately in each breast cancer subtype. Star shows where maximum AUCs were found.
