# Supplementary figures and images for: Effect of MR Imaging Contrast Thresholds on Prediction of Neoadjuvant Chemotherapy Response in Breast Cancer Subtypes: A Subgroup Analysis of the ACRIN 6657/I-SPY 1 TRIAL
Source: Tomography. 2016 Dec;2(4):378–87. doi: 10.18383/j.tom.2016.00247 (PMC5214452; doi:10.18383/j.tom.2016.00247)

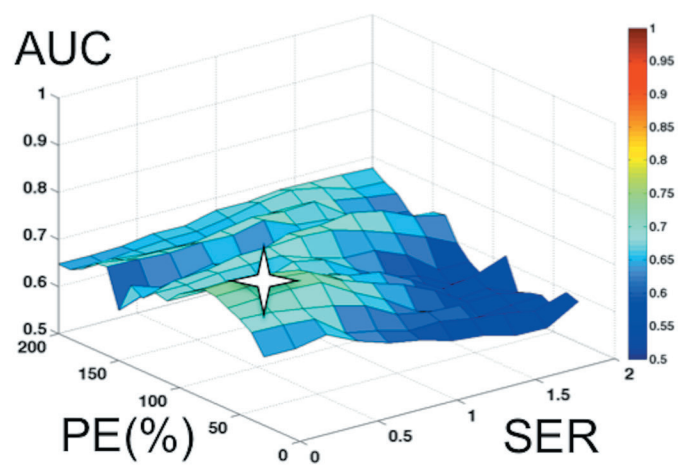

Full cohort

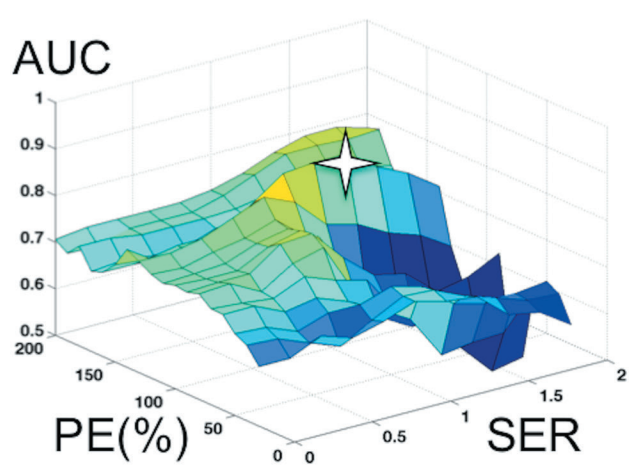

HR+/HER2-

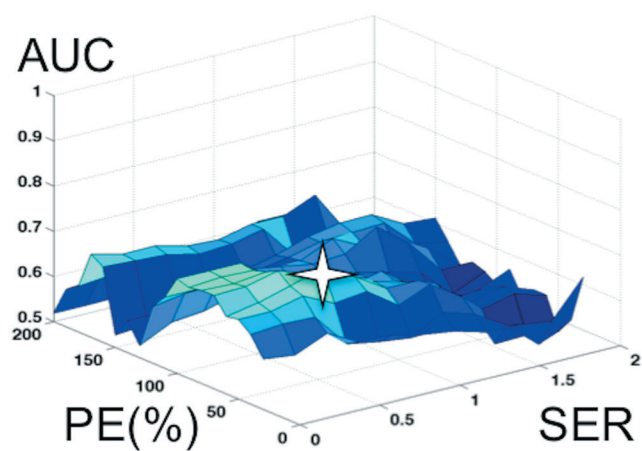

HER2+

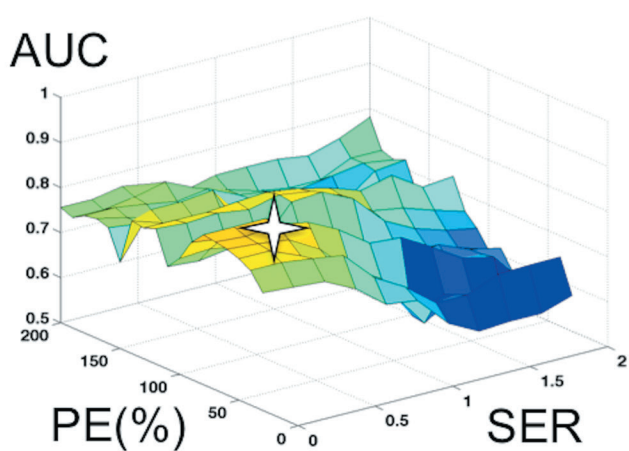

TNBC

**Figure S2.** Surface plots of estimated AUCs for  $\Delta FTV_3$  on  $PE_t/SER_t$  map.

Supplement: Supplemental Figure 2: [file tom-00247-16-s002.pdf]
