# Supplementary material for: Effect of MR Imaging Contrast Thresholds on Prediction of Neoadjuvant Chemotherapy Response in Breast Cancer Subtypes: A Subgroup Analysis of the ACRIN 6657/I-SPY 1 TRIAL
Source: Tomography. 2016 Dec;2(4):378–87. doi: 10.18383/j.tom.2016.00247 (PMC5214452; doi:10.18383/j.tom.2016.00247)
Supplement: Supplemental Figure 3: [file tom-00247-16-s003.pdf]

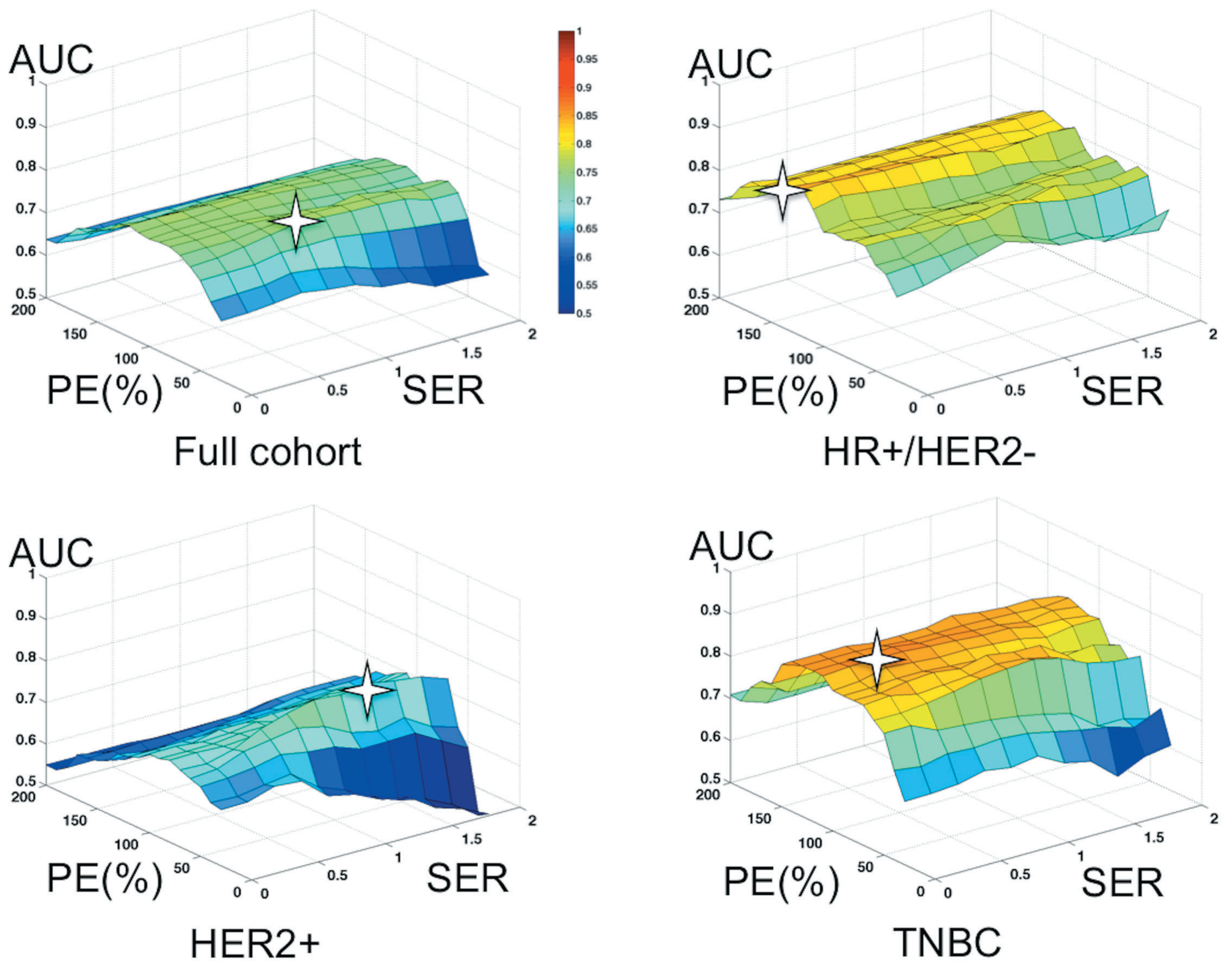

Figure S3. Surface plots of estimated AUCs for FTV_3 on PE_t/SER_t map.
